# Supplementary material for: Full-Length SMRT Transcriptome Sequencing and SSR Analysis of Bactrocera dorsalis (Hendel)
Source: Insects. 2021 Oct 14;12(10):938. doi: 10.3390/insects12100938 (PMC8537375; doi:10.3390/insects12100938)

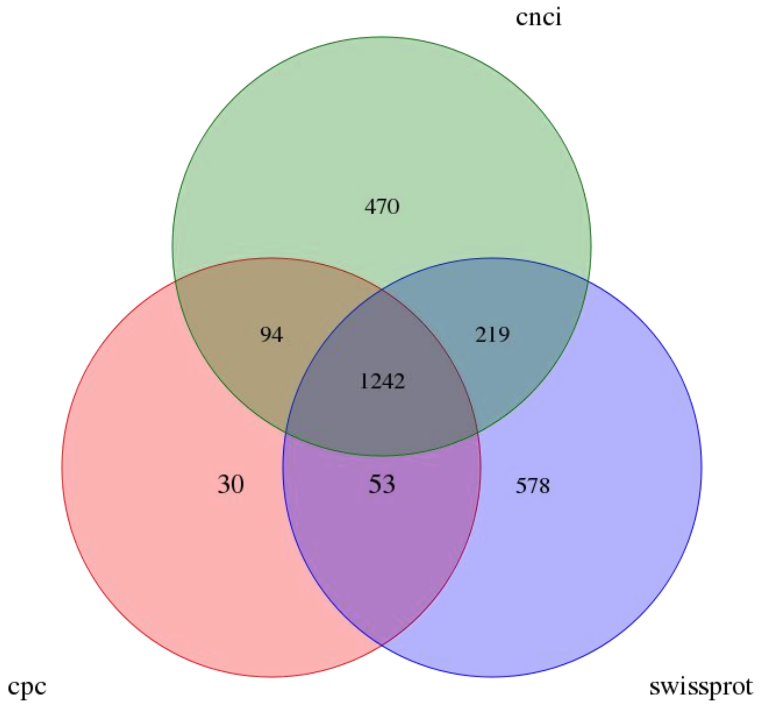

Figure S1. The Venn diagram of the number of lncRNAs predicted by CPC, CNCI and Swissprot.

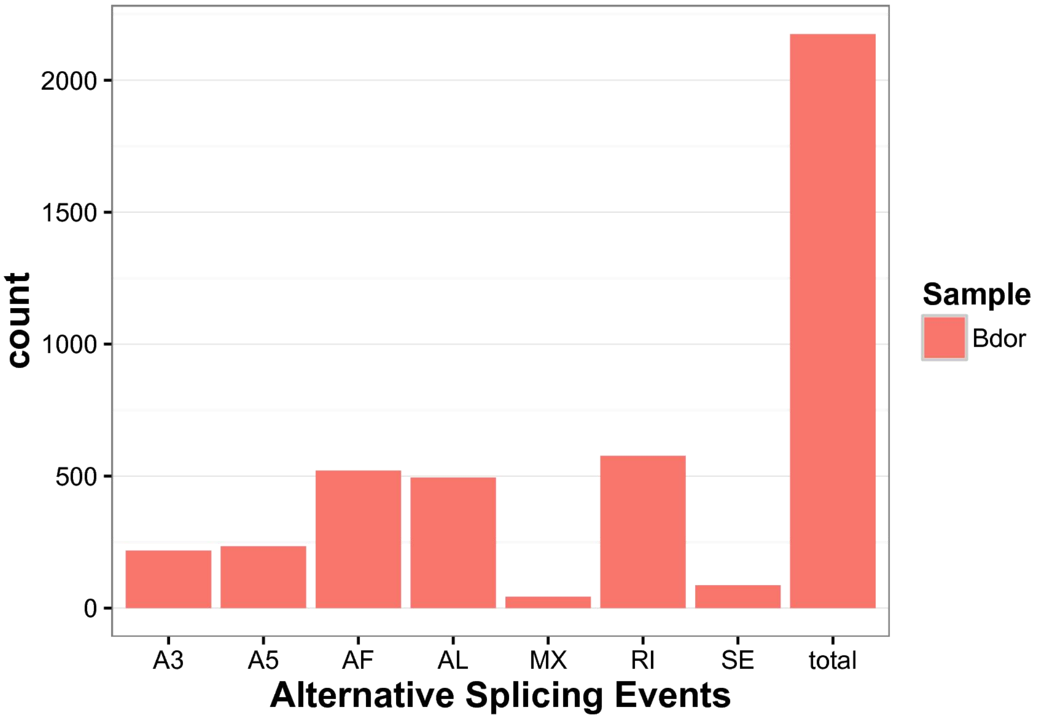

Figure S2. Classification and quantity statistics graph of alternative splicing events.

Table S1. Performance summary of 80 EST-SSRs with 174 *B. dorsalis* samples via nondenaturing polyacrylamide gel electrophoresis.

Classified information	Primer name	Electrophoretic diagram number
Ungenerated product	1114ACAT4	Picture 8
	3284GGC5	Picture 11
	4910ACA5	Picture 7
	4970AT6	Picture 7
	5405CATA4	Picture 20
Poor universal applicability	2904CTCA4	Picture 10
	3879TGG5	Picture 2
	4782GGTGGC4	Picture 7, 8
	5247AGGC4	Picture 6
	5452TATG4	Picture 6, 8
	5777GAT6	Picture 7
Multiple bands	2767TATG4	Picture 7
	4613CAG5	Picture 3
	4835ATAC4	Picture 4,5
Monomorphic bands	863CGC5	Picture 2, 12, 19-20
	1131TATG4	Picture 7
	1365TATG4	Picture 4,5
	2199ATTG4	Picture 3, 12
	2553ACAT4	Picture 21-24
	2923GGC5	Picture 11
	3150TACA4	Picture 3,4,6
	3533ACAT4	Picture 4,5
	3668TATG4	Picture 7, 29-31
	3923CAA5	Picture 7
	4756AAG6	Picture 4,5
	5769TA6	Picture 1,32
Unstable and unclear	559AAAT4	Picture 5
	1314CATA5	Picture 12,32
	1411CAA6	Picture 2, 9
	2170ATAC4	Picture 8
	3495ATGT4	Picture 8
	3721CAC6	Picture 8
	4434TATG4	Picture 10
	4771ACAT4	Picture 10
	4867TGA5	Picture 3, 11
	5346TCGC4	Picture 8
	5369TAA6	Picture 1,32
	5512CAA6	Picture 11
	5749TTAAT5	Picture 2
Stable and unclear	84GGC6	Picture 12
	1104AAC6	Picture 10

1807ATAC5	Picture 3, 12
2327TACA4	Picture 8
2402ATTT4	Picture 9
2859ACAT4	Picture 4,5
2922TACA4	Picture 7
2923CAG5	Picture 11
2923TACA4	Picture 7
2924TACA4	Picture 25-28
3194CAAGAG4	Picture 11
3393AGC5	Picture 11
3402AAAC4	Picture 10, 13
3695GCTCCA4	Picture 9
3866TATG4	Picture 8, 33-36
3954ACAT4	Picture 10
4367CAG5	Picture 7
4371ACAT4	Picture 6, 37-40
4461GCT6	Picture 10
4478AAC5	Picture 9
4488ATAC5	Picture 10
4513ATAC4	Picture 8, 20
4606CAG5	Picture 3, 12
4611CAG5	Picture 3, 12
4614CAG5	Picture 12
4621AT7	Picture 6
4621CAG5	Picture 2, 3, 11
4654TATG4	Picture 8
4707AC6	Picture 2, 10
4731ACAT4	Picture 4, 5
4777ATAC4	Picture 6, 8, 14-16
4932CATA4	Picture 32
4950CAA5	Picture 2, 9
4926TA7	Picture 1, 8, 17-18
4980CA7	Picture 3,6
4992CAA5	Picture 32
5040ATAC4	Picture 2, 9
5126TACA4	Picture 6
5257TAG5	Picture 3, 11
5749GCT5	Picture 1
5805AGC5	Picture 1, 9

Note: Referring to Figure S3 for electrophoretic diagram information.

Figure S3. Electrophoretogram of nondenaturing polyacrylamide gel.

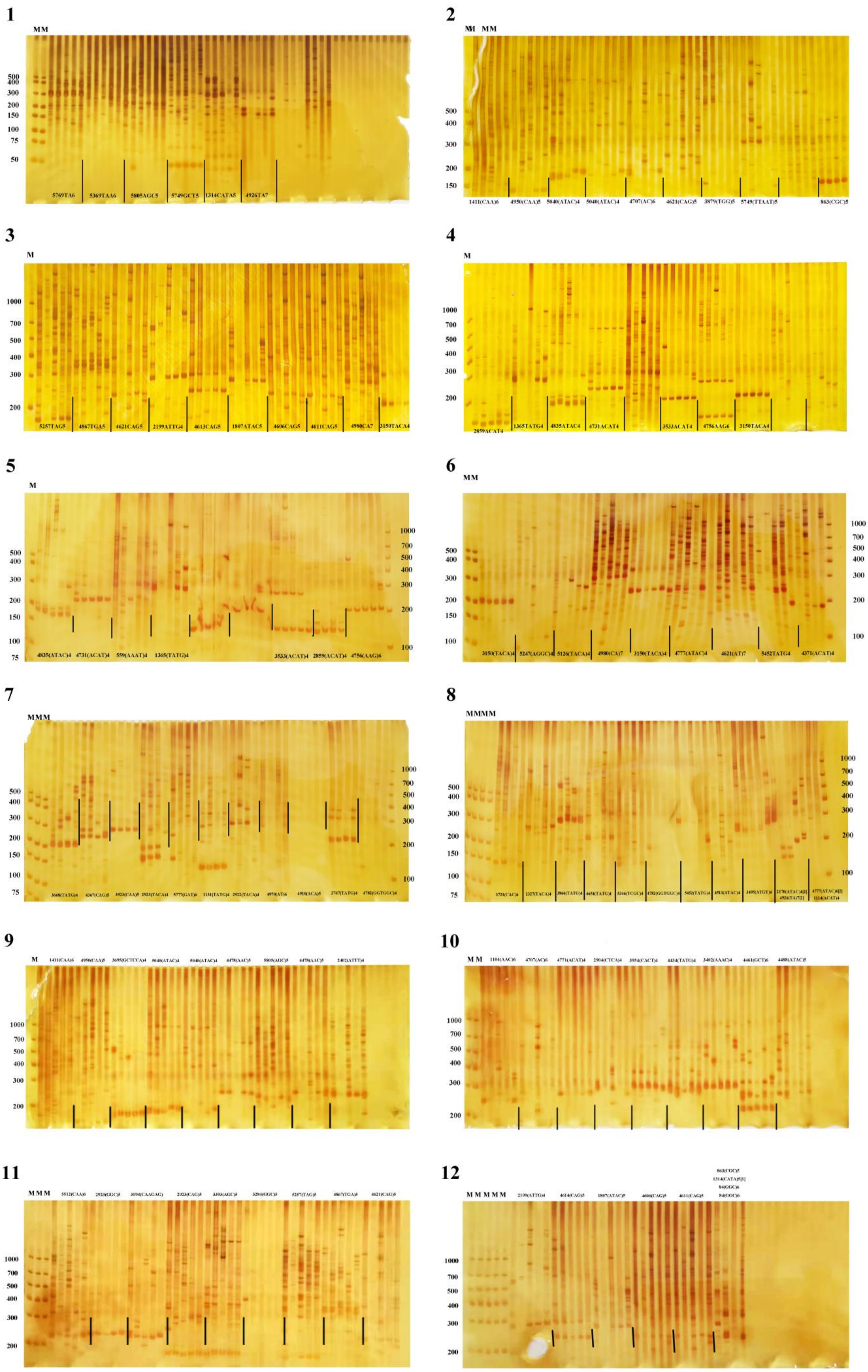

13

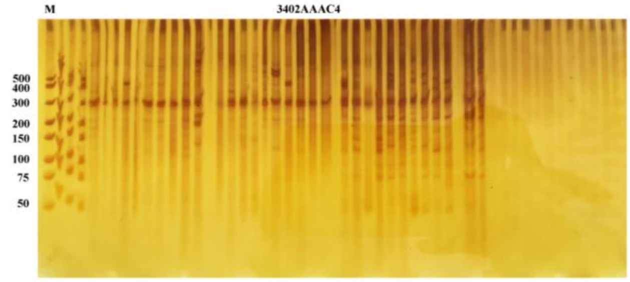

14

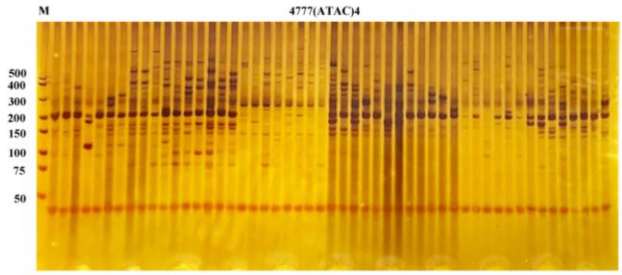

15

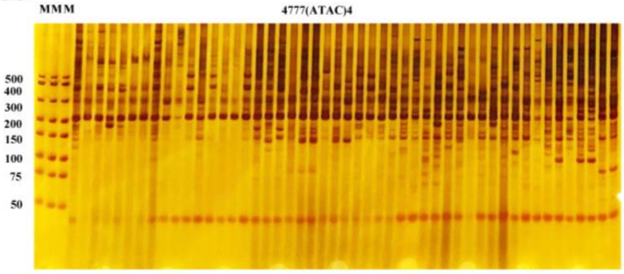

16

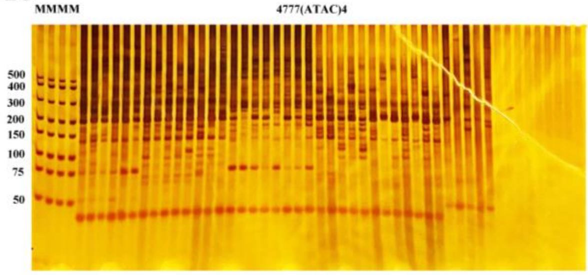

17

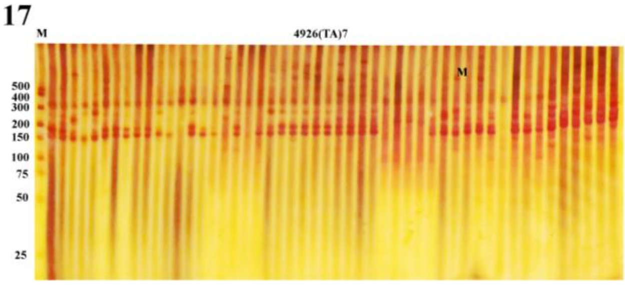

18

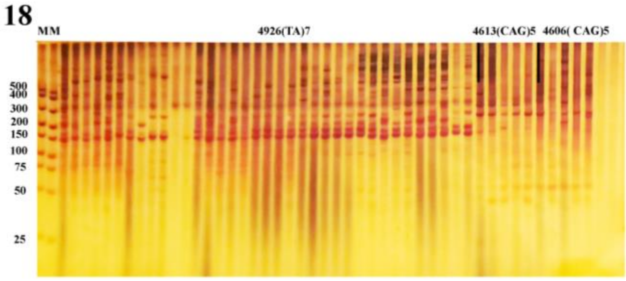

19

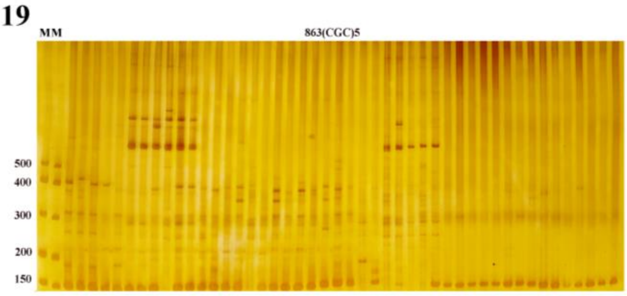

20

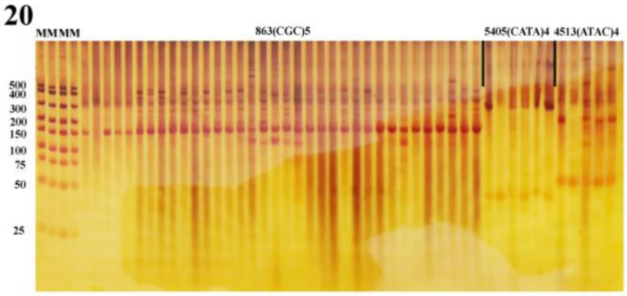

21

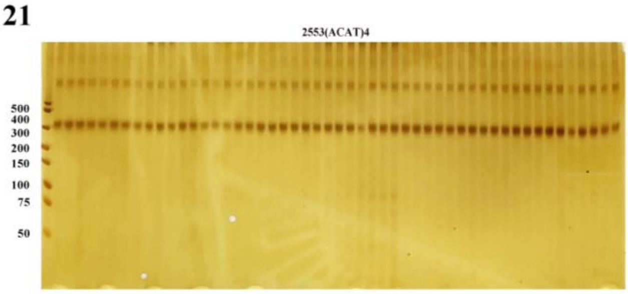

22

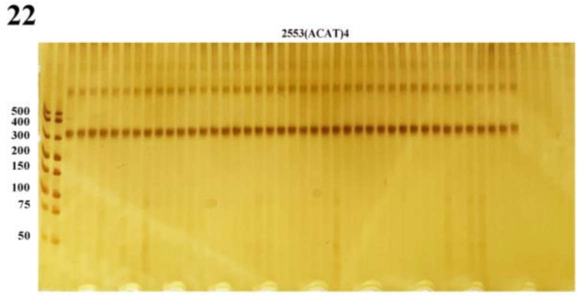

23

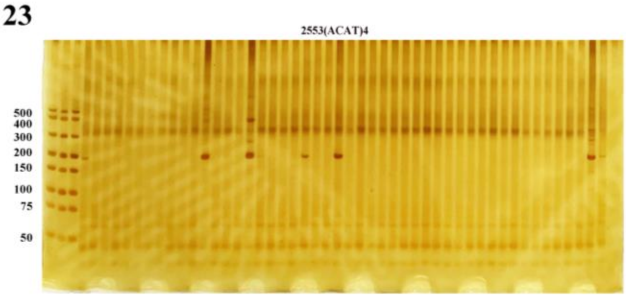

24

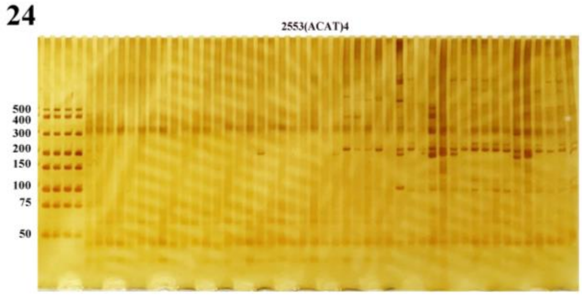

25

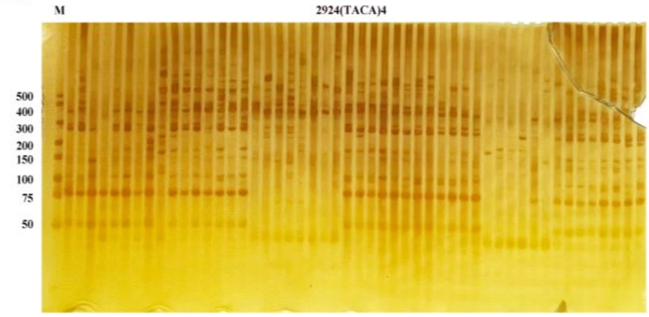

26

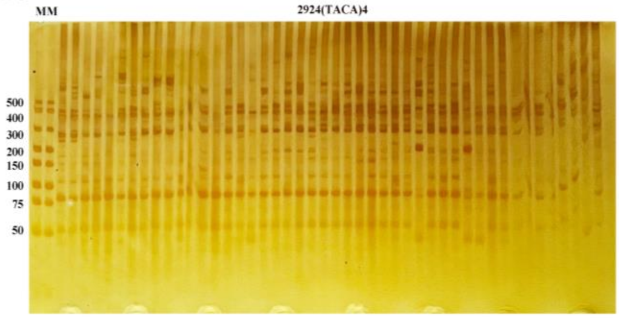

27

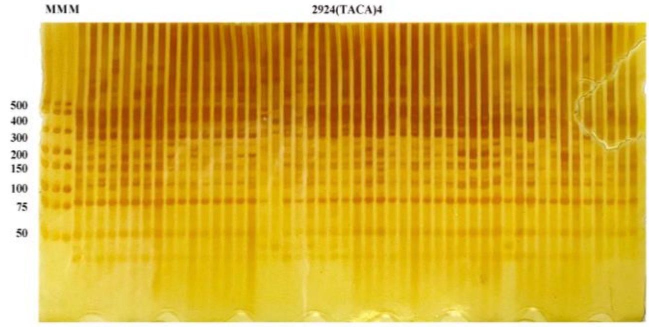

28

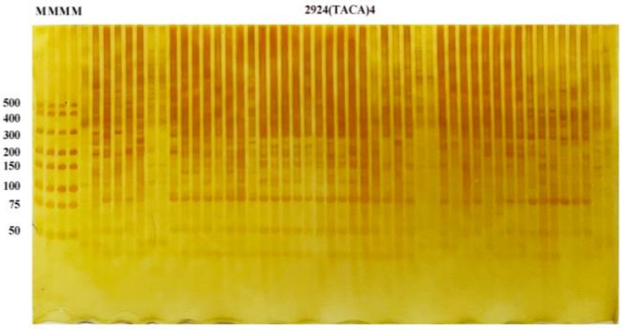

29

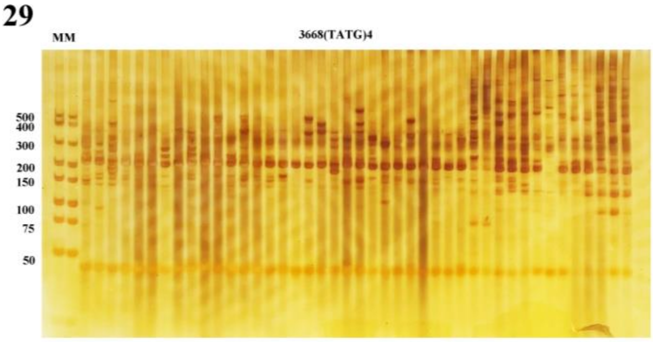

30

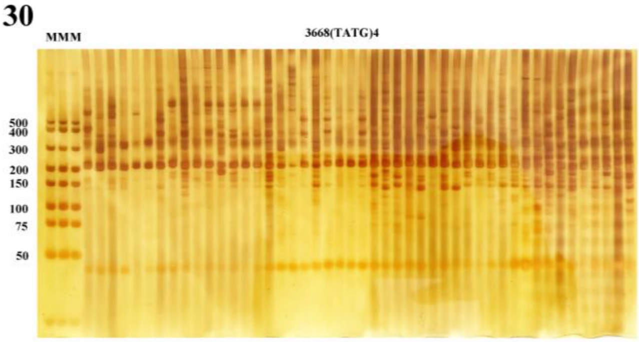

31

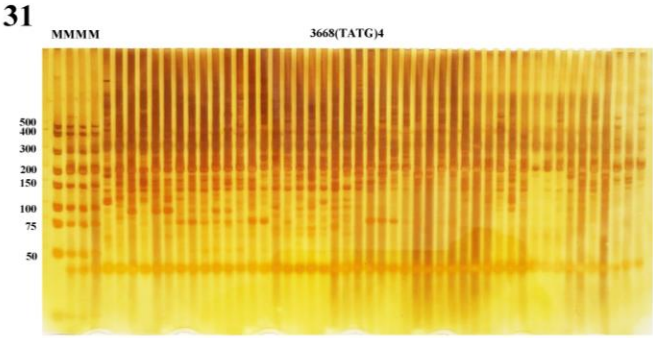

32

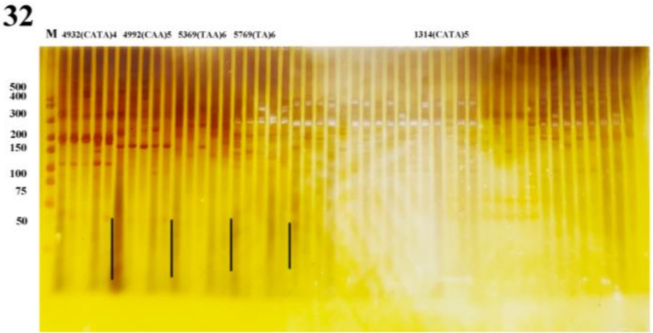

33

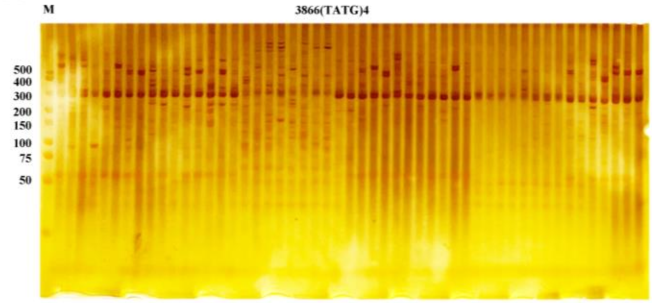

34

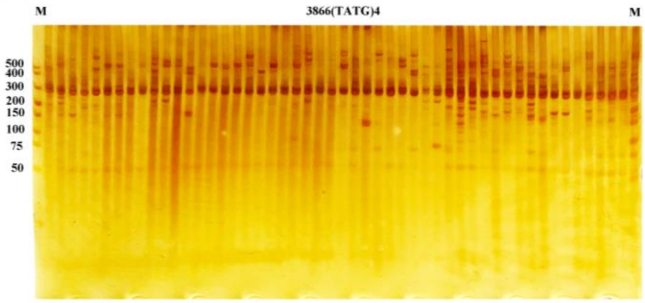

35

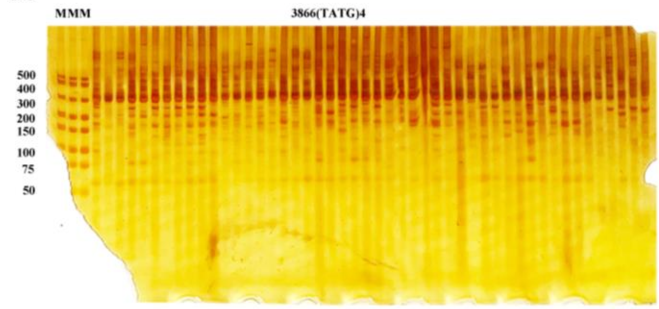

36

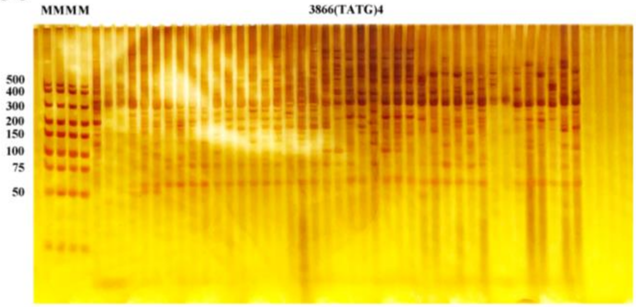

37

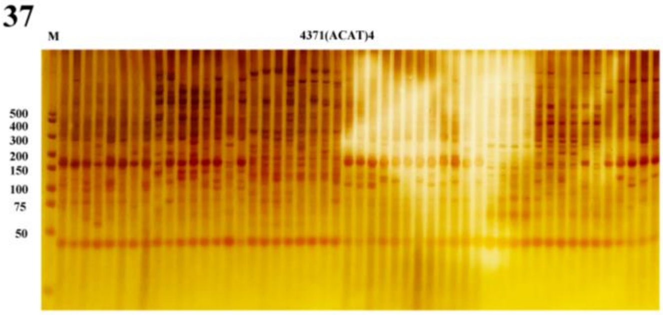

38

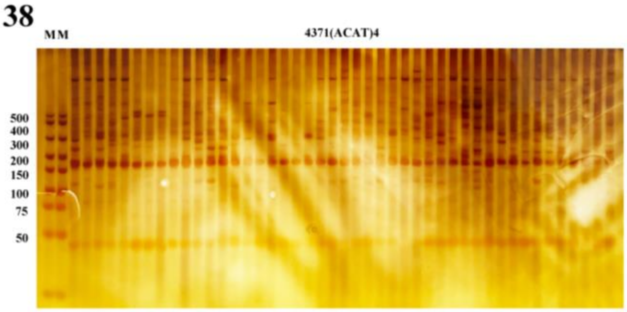

39

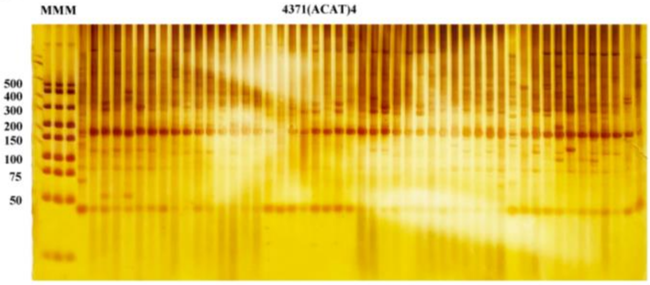

40

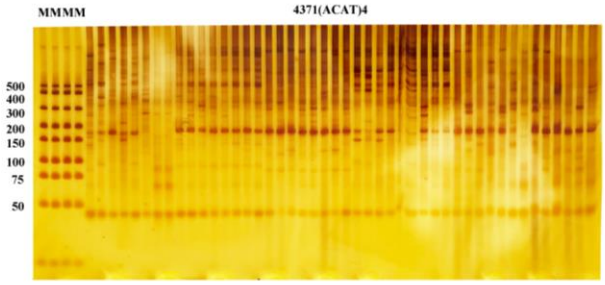

Supplement: Supplementary file 1 [file insects-12-00938-s001.zip › insects-1362783-supplementary.pdf]
